# Unpredictable, Counter-Intuitive Geoclimatic and Demographic Correlations of COVID-19 Spread Rates

**DOI:** 10.3390/biology10070623

**Published:** 2021-07-05

**Authors:** Hervé Seligmann, Nicolas Vuillerme, Jacques Demongeot

**Affiliations:** 1 Laboratory AGEIS EA 7407, Team Tools for e-Gnosis Medical & Labcom CNRS/UGA/OrangeLabs Telecom4Health, Faculty of Medicine, University Grenoble Alpes (UGA), 38700 La Tronche, France; Nicolas.Vuillerme@univ-grenoble-alpes.fr; 2 The National Natural History Collections, The Hebrew University of Jerusalem, Jerusalem 91404, Israel; Jacques.Demongeot@univ-grenoble-alpes.fr

**Keywords:** exponential regression, SARS-CoV-2, contagiousness, pandemic, orthoevolution, negative heritability

## Abstract

**Simple Summary:**

Rates of viral spread during first and second waves of the COVID-19 pandemic for USA states, and for consecutive nonoverlapping periods of 20 days for the USA and 51 countries across the globe associate with mean temperature, elevation, population density and age. Some associations switch directions when comparing different periods. Even population density, which presumably should always increase viral spread, at some periods seems to decrease spread rates. We also observed systematic inversions between spread rates estimated at 80–100 day intervals. These patterns remain unexplained and suggest difficulties in managing and predicting the pandemic, in particular, negative correlations between population density and spread rates, which were observed in independent samples and at different periods. Putatively, confinements could produce these patterns, by selecting viral strains with longer contagiousness and/or latent periods.

**Abstract:**

We present spread parameters for first and second waves of the COVID-19 pandemic for USA states, and for consecutive nonoverlapping periods of 20 days for the USA and 51 countries across the globe. We studied spread rates in the USA states and 51 countries, and analyzed associations between spread rates at different periods, and with temperature, elevation, population density and age. USA first/second wave spread rates increase/decrease with population density, and are uncorrelated with temperature and median population age. Spread rates are systematically inversely proportional to those estimated 80–100 days later. Ascending/descending phases of the same wave only partially explain this. Directions of correlations with factors such as temperature and median age flip. Changes in environmental trends of the COVID-19 pandemic remain unpredictable; predictions based on classical epidemiological knowledge are highly uncertain. Negative associations between population density and spread rates, observed in independent samples and at different periods, are most surprising. We suggest that systematic negative associations between spread rates 80–100 days apart could result from confinements selecting for greater contagiousness, a potential double-edged sword effect of confinements.

## 1. Introduction

Spread parameters of daily new confirmed COVID-19 cases (calculated as the slope of their logarithmic regression curve) estimates viral contagiousness. Previously, it was shown that first wave spread, when comparing different countries, decreases with mean annual temperature [1], and the opposite trend with temperature occurs for second wave spread parameters [2].

This is in line with observations on variation in spread across different regions of Italy, in March 2020 (first wave period, negative correlation with temperature) and in May 2020 (second wave period, positive correlation with temperature) [3].

First and second wave spreads differ also in terms of other factors: The spread of the first wave increases with the median population age and decreases the later the date of its onset. On the contrary, the spread of the second wave decreases with the median population age and increases the later the date of its onset. First and second waves also differ because we detected no associations between second wave slopes and mean country elevation, while first wave slopes increase with elevation up to 900m and decrease beyond that approximate altitude [2].

Inversion of trends for these independent covariates is difficult to explain. One could invoke different explanations for each factor. A common, most parsimonious explanation could involve deterministic mutation dynamics, resulting in parallel evolution of distinct virus populations [4,5].

This working hypothesis expects cycles in the epidemiological behavior of the virus, where pattern inversions occur each time a sufficient number of mutations cumulated to cause a switch in secondary structure of the single-stranded RNA coronavirus. This would explain the inversion in patterns between first and second waves. Because mutations cumulate on average proportionally to time, one expects that epidemiological pattern inversions occur after a fixed time interval.

Here, we calculate spread rates for daily new confirmed COVID-19 cases for consecutive, nonoverlapping time windows of 20 days since the start of the pandemic in 51 different countries, and in each of the 50 states of the USA (and the district of Columbia) until the end of 2020, and examine these spread rates for inversions in patterns of correlations with some environmental factors (temperature, elevation) and population properties (density, median age), and across spread rates for different time windows. This approach, which calculates spread rates for each consecutive period of 20 days, avoids difficulties inherent to the objective definition of the start and end of different pandemic waves.

## 2. Materials and Methods

We used the methods as in previous analyses [1,2]. The coefficients (slopes) of regression analyses are considered as estimates of viral spread rates. We adjust the exponential model y = a × exp(b × x), where y is the daily number of new confirmed COVID-19 cases, x is the number of days since wave onset, a is a constant and b is the slope. The log-transformed version of this is ln y = ln a + b × x. Daily numbers of new cases per countries are from the site www.worldometers.info/coronavirus/ (accessed on 25 December 2020).

Spread rates are determined for running windows of 20 consecutive days, where consecutive time windows are nonoverlapping. Hence, if the first new case in a country occurred 280 days ago, 14 spread rates were calculated, one for each of the nonoverlapping 20-day periods. Spread rates are estimated for these running windows as described above, the slope of the exponential model of new daily cases with time.

For countries, sources for mean elevation, mean temperature, population density and median population age and numbers of genomic variants are as previously described [2]. For the 50 states of the USA, we used the following sources in Table 1:

We also analyze daily new confirmed case data for each of the 50 states of the USA and the district of Colombia, estimating first and second wave spread rates as done previously for countries across the globe [1,2], using for the USA the exact same methods as described previously [1,2].

Note that the slopes we evaluate from daily data in daily new cases estimate the acceleration in increase of new cases: in the first differential model proposed for the COVID-19 outbreak [6], the variable is the number of cumulative cases at time t, denoted X_t_, the velocity is the number of daily new cases, denoted Y_t_ = (X_t_ − X_t − 1_)/(t − (t − 1)), and the acceleration is the slope of the curve of these daily new cases, denoted Z_t_ = (Y_t_ − Y_t − 1_)/(t − (t − 1)). This acceleration as observed in simulation of the differential model, has a first phase where daily new cases grow exponentially, until an inflection point is reached with acceleration zero before the saturation phase.

We tested for normality of the distribution of variables used in correlation tests with the Kolmogorov–Smirnov test, as available at Kolmogorov–Smirnov Calculator (Test of Normality) (socscistatistics.com, accessed 20 December 2020). For variables statistically significantly diverging from normality (*p* < 0.05), we calculated both parametric Pearson and nonparametric Spearman rank correlation coefficients at Spearman’s Rho Calculator (Correlation Coefficient) (socscistatistics.com, accessed on 20 December 2020)).

## 3. Results

### 3.1. Mutations Cumulate over Time during the COVID-19 Outbreak

**Figure 1:**
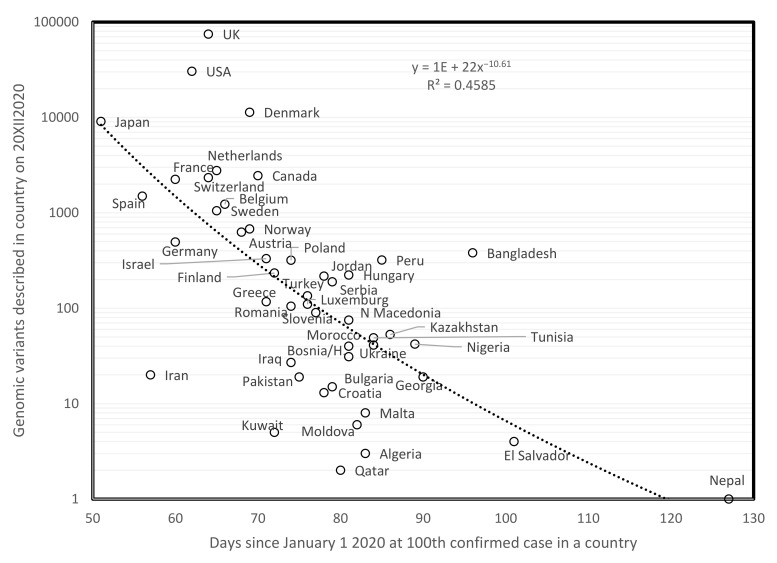
Numbers of genomic variants of SARS-COVID-19 as a function of days since 1 January 2020 until 100 confirmed cases were detected in a country.

Figure 1 plots the total number of genomic variants described for a country since the start of the pandemic, on 20 December 2020, as a function of the numbers of days since 1 January 2020 when the cumulated number of confirmed cases in a country reached 100 cases. This method for estimating the duration of the pandemic is not adequate for states/countries for which the pandemic presumably stopped before end 2020. These are excluded from analyses. Overall, the longer the pandemic is ongoing in a country (few days since 1st of January), the more mutants have been described in that country. The negative correlation confirms that numbers of mutations are proportional to time. A similar result was found on 31 May 2020 [2]. Hence, this phenomenon is stable over time. 

It is probable that numbers of variants reported from a country reflect also the sequencing efforts in that country, which vary among countries. However, the association in Figure 1 is too strong to be only due to this factor, and probably reflects also, or even more, an actual natural cumulation of mutant variants. Indeed, along that rationale, population size should also increase variant numbers, but no such effect is observed, as small and large countries fit equally well the curve.

### 3.2. Spread Parameters for First and Second Waves in the USA

Table 2 presents for each of the 50 states of the USA onset dates and slopes of first and second waves, as detected by visual inspection as previously [2]. At mid-August, 42 among 50 states have a second wave. Second wave slopes are lower than first wave slopes in all but three states: Oklahoma (slopes basically identical), and Kansas and Ohio (second wave spread greater than first wave spread).

Hence, overall, spread decreased from the first to the second wave, from mean first wave slope = 0.1902 to mean second wave slope = 0.0585.

In the USA, first wave slopes decrease with time since first wave onset (r = −0.5306, two-tailed *p* = 0.000074, Spearman rank correlation coefficient rs = 0.415, *p* = 0.003). This is similar to the decrease in slopes previously reported for comparisons across countries [2]. Comparing countries, second wave slopes increase with time since second wave onset [2]. However, for the USA, no such increase could be detected, the trend might fit with that observed for the first wave (r = −0.29, *p* = 0.06, Spearman rank correlation coefficient rs = −0.365, *p* = 0.019). Also contrasting with previous analyses of variation between countries, there is no association in the USA between first and second wave slopes and mean annual temperature. A positive association exists between first wave slope and time since second wave onset (r = 0.4469, two-tailed *p* = 0.003), which could indicate that high initial spread rates contribute to early onset of ulterior waves. Note that this effect does not explain the absence of second waves in several states with particularly high first waves, such as Illinois, New Jersey and New York.

**Figure 2:**
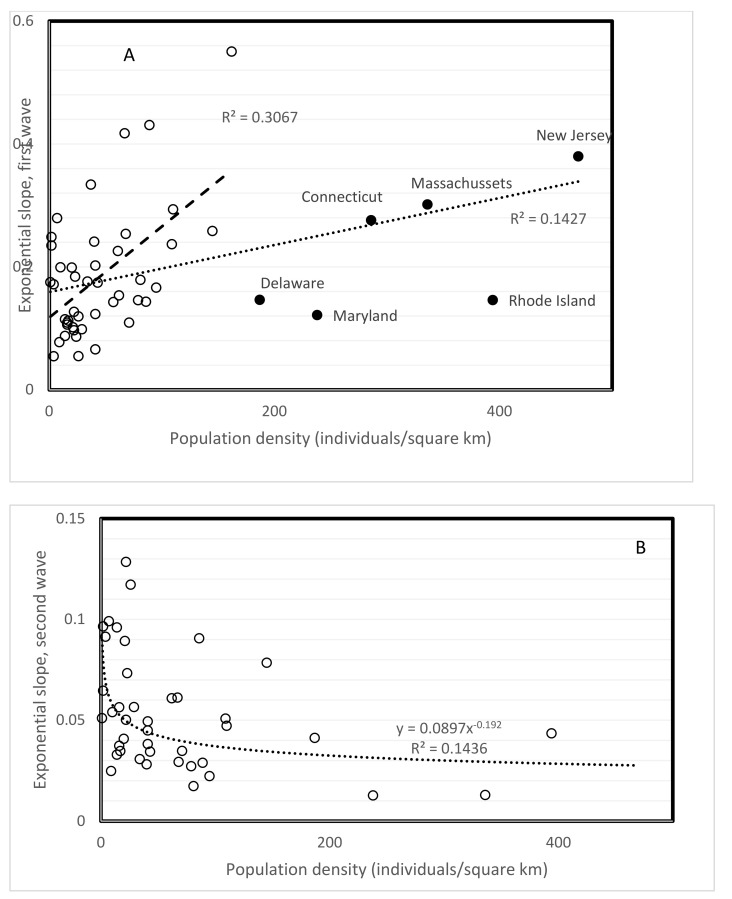
Spread parameters (slope of exponential regression) of first (**A**) and second (**B**) waves in USA states as a function of population density. Trend inverted between first and second wave. A: the New England states with high densities (filled circles) follow a different pattern than the rest of states, with a slower increase in spread with increasing density. B: the second wave slopes (as determined in August 2020) are overall lower than first wave slopes, and counter-intuitively decrease with population density. Patterns remain statistically significant with nonparametric Spearman rank correlation tests, see text.

First wave slopes increase with population density (r = 0.3777, one-tailed *p* = 0.0034, Figure 2A). This association is expected, but was not observed when comparing different countries [1,2]. Six New England states have relatively low slopes considering their densities. Excluding them from calculations increases the strength of this correlation with density (r = 0.5538, one-tailed *p* = 0.00005).

Density in principle increases spread rate. This was not observed for first and second wave slopes when comparing countries [1,2]. However, this density principle is confirmed for first wave slopes from the USA (Figure 2A). Unexpectedly, second wave slopes decrease with population density (r = −0.459, two-tailed *p* = 0.0022, Figure 2B). Unexplained pattern inversions between first and second waves have been reported for covariates such as temperature, time since wave onset and median population age when comparing different countries [2]. Decrease of slopes with density in the USA is foremost astounding because it does not at all fit with any knowledge on disease spread.

### 3.3. Visual Inspection vs. Objective Statistical Analysis

Arguably, determining onsets of waves from visual examinations of graphs is subjective. This issue has been raised in the past [7,8], but has no obvious simple solutions and requires extensive simulation analyses curtailed to each specific dataset, meaning in this case for each state [9,10]. We therefore use a simplified method. Statistical analyses based on calculating Pearson correlation coefficients r for a running window of 20 days determine a local maximum for r within five days of the second wave onset date determined visually for 80 percent of the countries examined [2]. We applied this method for the 42 second waves detected for the USA (Dis, Table 2). Most of the onset dates determined by running windows (62%) are within five days of the date determined visually, in line with similar previous analyses for other countries [2]. The spread rates calculated for nonoverlapping, consecutive windows of 20 days presented below do not suffer difficulties inherent to defining wave onset.

### 3.4. Spread Rates for Consecutive Nonoverlapping 20-Day Windows

We estimated spread rates for 14 and 15 consecutive, nonoverlapping 20-day periods for the 50 states of the USA and the district of Colombia (Table 3), and 51 countries across the globe (Table 4). Note that because the start of the pandemic varies among states and countries, spread rates are not exactly for the same period when comparing states/countries. Numbers of states and countries where rates are positive are highest for the earliest period (approximately end of February to beginning of March, day 20). This number is lowest during the warmer spring and summer months. This is in line with an overall decrease of spread with high temperatures.

Table 5 presents the pairwise Pearson correlation coefficients for the spread rate data from Table 3 and Table 4 (USA, above diagonal, data from Table 3; countries across the globe, below diagonal, data from Table 4). These 91 and 105 r’s include 16 cases (eight cases for the USA) where the correlation between spread rates for two periods are positive and statistically significant (*p* < 0.05, 2-tailed tests), and 14 and 19 cases, respectively, where r is negative and statistically significant (*p* < 0.05, 2-tailed tests). Among the 16 statistically significant positive cases, a statistically significant majority, 13, are between consecutive periods (two-tailed sign test, *p* = 0.011). This indicates that spread rates are relatively similar across two consecutive periods of 20 days, hence for about 40 days.

**Figure 3:**
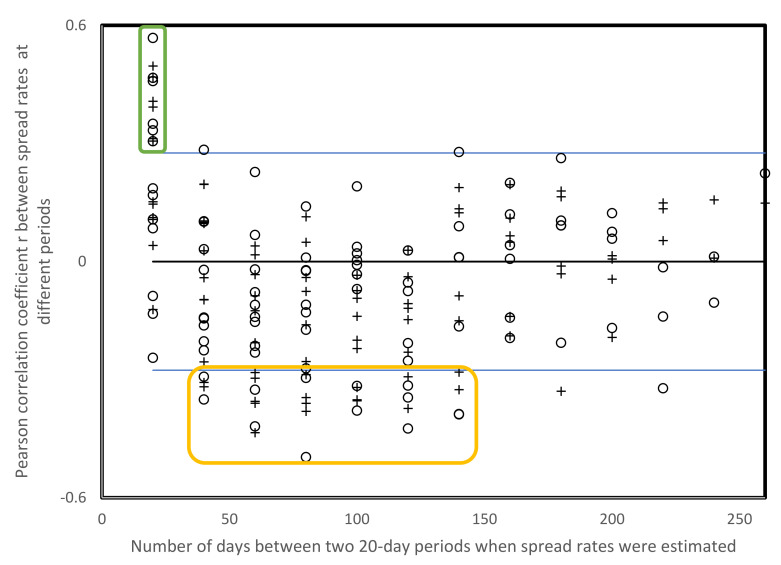
Pearson correlation coefficients r between spread rates from Table 3 and Table 4 (open cirles, USA; and +, other countries, r from Table 5) as a function of numbers of days between the time frames when spread rates were estimated. Blue lines indicate crirical values of r for *p* < 0.05 according to two-tailed tests. Most positive r’s with *p* < 0.05 are for pairs of consecutive periods (20 days on *x* axis, green box), most negative r’s with *p* < 0.05 are for periods separated by approximately 80 days, orange box.3.5.

The distribution of statistically significant negative correlations among spread rates from Table 3 and Table 4 in relation to time periods is greater than for positive correlations, but 13 among 14 cases are for periods separated by 40–140 days, on average 88 days with a standard deviation of 30 days (Figure 3). Two statistically significant negative correlations between spread rates are separated by 160–240 days.

### 3.5. Environmental Correlates of Spread Rates across Time

Table 6 presents Pearson correlation coefficients r between spread rates in Table 3 and Table 4 and environmental variables from Table 2 and Table 7. Results include inversions of directions of correlations, as also shown in Table 5 and Figure 3. For mean annual temperatures, around May (day 100), spread rates increase with temperature in the USA and in the world, as previously reported for countries across the world [2], and for the period of December (day 280, USA only). Temperature decreases spread rates at the end of summer and beginning of autumn in the USA and across the world. Results for mean altitudes show similar pattern inversions, including inversion between consecutive periods at days 180 vs. 200 (August vs. start of September, USA only). For the USA, an inversion in correlation directions also occurs with median age, with positive vs. negative statistically significant correlations at days 180 vs. 200. The negative correlation of spread rates with age corresponds to the time when schools open (end of August to beginning of September). Spread rates increase again with median age at the end of autumn (day 280, first part of December). For countries across the world, median age increases with spread rates at pandemic onset, and in summer–autumn, for the period spanning from day 160 to 240. In contrast, for the spring periods from days 60 to 100, spread rates decrease with median age when comparing countries across the world. This might result from seasonal differences between age-related behaviors, but other explanations are also possible.

The only statistically significant correlations with population density when comparing countries across the world are detected using nonparametric Spearman rank correlation analyses, with the only positive associations fitting trivial expectation on consecutive periods spanning days 180 and 200 (*p* < 0.05, one-tailed tests). Density is expected to increase spread rates. Such a positive association is observed at pandemic onset in the USA. This pattern is inversed for three consecutive periods in the spring in the USA and at days 80 and 280 for countries across the world.

Negative associations of viral spread rate with density are unexpected and make no sense in terms of classical epidemiological understanding, as these imply greater spread rates in populations with low density.

## 4. Discussion

### 4.1. A Potential Epidemiological Explanation for Spread Rate Inversions

The inversion of patterns with time and other variables (altitude, temperature, median population age) for slopes of pandemic waves renders predictions particularly dubious. In addition, the negative correlation between second wave slopes and USA density (Figure 2B) contradicts a proven and accepted epidemiological principle. The inversion of directions of correlations between spread rates and several environmental factors (Table 6) confirms previous observations regarding temperature [2]. Periods until inversions vary among environmental factors. For example, associations of spread rates with age flip directions from increasing to decreasing with median age at the transition between summer vacations and the beginning of the school year, at least in the USA. One could find for each of the inversions in directions in statistically significant r values in Table 6 a specific explanation, such as that stated above about school openings. However, the almost systematic inversion of correlation directions for most environmental factors suggests a common cause for most of these pattern inversions.

This point for a more general cause for pattern inversions is strengthened by correlations between spread rates at different periods, for USA states and for countries across the world. The spread rate hierarchy among US states and among countries is systematically inverted after 80–90 days (Table 4 and Figure 3). The cause for these inversions is unclear, but this inversion pattern is systematic, as spread rates at any period will be inversely proportional to those at an ulterior time, typically 80–90 days later. Note that 80–90-day intervals between slopes are too long for these slopes to be part of the ascending and descending parts of the same wave, as proven in the next section.

A first potential explanation is that spread rates vary according to cycles of maxima, intermediate and minima in daily case numbers, and that countries with the highest maxima have the lowest minima and those with the lowest maxima have the highest minima, about 80–100 days later. This hypothesis assumes that the same rules also apply to periods with intermediate spread rates, and that ups and downs in spread rates are synchronized between most countries. The latter assumption is adequate for the first wave in late winter 2020, but could not hold throughout the complete period analyzed, also because countries and states vary in confinement periods, lengths and efficiencies, which causes decreases in spread rates. Hence, additional analyses are required to understand the causes of these pattern inversions; however, the systematic aspect of these inversions in spread rates is such that these should be accounted in the predictions and policymaking. This is because results of correlation analyses hint at the possibility that steep decreases in spread rates cause steep ulterior increases. In that respect, it might be optimal to mitigate variations in spread rates by avoiding drastic policies suddenly increasing or decreasing spread rates.

### 4.2. Negative Correlations between Spread Rates Are Not between Ascending and Descending Parts of the Same Wave

We test here the hypothesis that negative correlations between spread rates from different periods reflect negative associations between the steepness of the rate during the ascending and the descending parts of the same wave can be tested. This is easily tested using data in Table 3 and Table 4, because spread rates in the ascending phase of a wave are positive, and those in the descending phase of a wave are by definition negative. So, if the hypothesis is correct, for statistically significant negative correlations in Table 5, we should see systematically the opposite sign for rates in the two periods considered. If this inversion of signs is not observed, the ascending/descending phase hypothesis is incorrect.

We tested this for each of the negative correlations with *p* < 0.05 in Table 5, as presented below for a specific example. We considered the negative association between spread rates for the USA between periods of 20 days starting at days 100 and 240 after the start of the outbreak. For these periods, among the 51 states and district of Colombia, there are 26 and 48 positive spread rates. Hence, the probability of random assortment of identical signs between these two periods expects 26 × 48/51 + 25 × 3/51 = 25.94 where spread rates in the two periods have the same sign, and 51 – 25.94 = 25.06 cases where signs are inverted between the two periods. The observed number of inverted signs between these periods are 28. This slight increase as compared to the expected has *p* = 0.41 according to a chi-square test.

This analysis was done for all negative correlations with *p* < 0.05 in Table 5. There are 10 (USA, 5; countries across the world, 5) negative associations between spread rates where sign inversion between periods is significantly greater than random predictions, and 23 cases where this effect is not statistically significant as in the above example. This means that the hypothesis of ascending/descending phases of a wave could contribute in some cases to the phenomenon of negative associations observed between spread rates from different periods, but is not the main cause of these negative associations.

### 4.3. Does Confinement Increase Ulterior Spread Rates?

**Figure 4:**
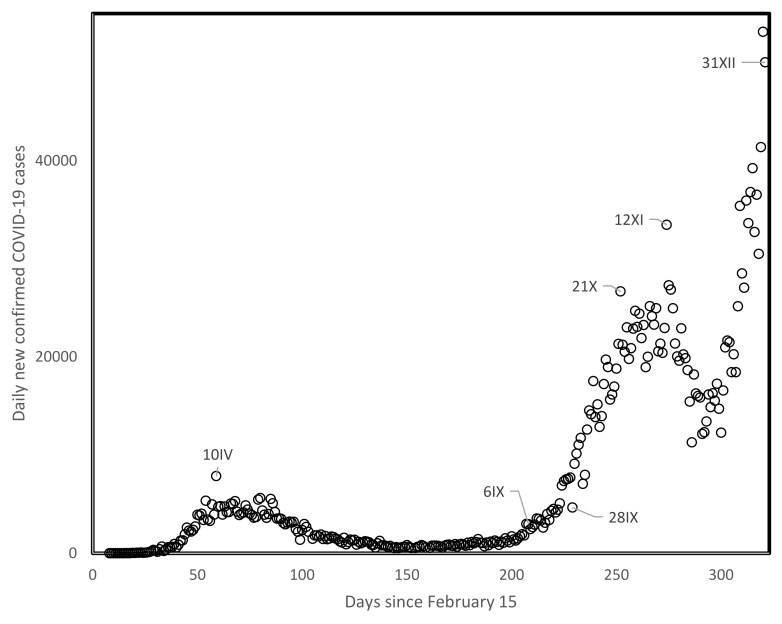
Daily new confirmed COVID-19 cases in the UK since 15 February 2020 until the end of 2020. The dates, with months counted in roman numbers are indicated for specific datapoints.

One mechanism that could be invoked in this context is that strict confinement policies select for highly contagious viral strains and/or long contagion periods that are more likely to overcome confined conditions. As case numbers decrease during confinements, reopening societies to normal activity unleashes these more contagious viral strains while potentially competing strains with lower infection abilities disappeared, resulting in higher spread rates at the level of the whole population. This dynamic would presumably be visible at the level of analyses such as those done here at time lags of 80–90 days, and would presumably cause the systematic inversions in spread rates among countries and regions described here. Note that the natural trajectory of viral evolution typically increases contagiousness while at the same time decreases pathogenicity, also in the absence of confinement. Confinements hasten the process of evolving greater contagiousness. Steep increases in recent months (autumn of 2020) in the UK, apparently associated with a new highly contagious viral variant, seem in line with this working hypothesis (Figure 4).

A coolheaded analysis and evaluation of above and future results is crucial for better managing the pandemic.

### 4.4. Intrinsic vs. Extrinsic Constraints

We discuss below additional potential, more speculative and genetics-oriented explanations for inversions of directions of correlations with spread rates.

One hypothesis is that self-hybridization of the virus’ single-stranded RNA genome protects nucleotides forming stems against mutations, while favoring mutations in loops [11]. Mutation cumulation presumably causes deterministic switches between few optimal structures which differ in their properties in relation to temperature, etc. Such switches between secondary structures have been suggested for COVID-19 after small insertion/deletion mutations [12].

These patterns remind the little-known phenomenon of negative heritability. Usually, heritabilities of traits are such that offsprings resemble their parents: if a parent is for a given trait above average, his/her offspring will on average be also above average, and parents below average for that trait have on average offspring which are below average for that trait. In short, heritabilities are correlations between traits of parents and offspring. These correlations are usually positive. Surprisingly, in some cases, negative correlations were observed, meaning negative heritabilities, where above average parents produce below average offspring for given traits, and vice versa for below average parents [13,14]. These phenomena are not statistical artefacts [15] but are difficult to reproduce empirically [16]. This is expected when assuming negative heritability results from selection under changing environmental conditions [17].

Inversion of trends, such as for negative heritabilities, could presumably result from drastic environmental changes. For example, levels of channeling of *Sorghum bicolor* plant populations towards developmental trajectories better adapted to NaCl salinity after early low level NaCl exposure increased with population variability [18] in the first generation exposed to salinity, but decreased with populational variability in their offspring [19,20]. This is in line with the concept that COVID-19 adapts to its new human host and to various environments inhabited by that host.

It is possible that a large part of the variation in slopes during the pandemic is due to factors intrinsic to evolutionary trajectories of the virus, genetic ones included. This does not exclude environmental effects due to host population age structure, density, temperature and altitude, among others. However, inversions of trends with these cofactors at different periods of the pandemic suggest that unknown intrinsic factors have major effects on the evolution of the pandemic.

Another approach stipulates that variations across different locations reflect to large extents the dynamics at a single location, at different times. This principle where spatial variation is tantamount to temporal variation has been observed in many different contexts, such as in astrophysics where larger distances are interpreted as reflecting more ancient times [21], and in forest stage succession [22,23,24], ecological communities [25] and biomolecules such as those involved in ribosomes [26,27,28,29,30,31]. The principle is also applied in the renown Haeckelian statement “ontogeny recapitulates phylogeny” [32,33]. Variation among individuals in directional asymmetry is interpreted as reflecting different termination timings of development [34]. The principle exists within the relationship of the genetic code and the ribosome [35]. Similarly, the average order of translation of amino acids in single proteins reflects the order of integration of amino acids in the genetic code [36]. At the level of the pandemic, this would suggest that the macroscopic spread rates correspond to viral replication cycles and/or rates of production of viral particles. This hypothesis should be considered as suggestive at best, but could be proven useful as a working hypothesis for the long-term dynamics of this still unknown disease at the level of individuals.

Pattern inversions add to the problem of uncertainty in the data and in predictions [37,38]. Analyses of spread rates estimated for consecutive nonoverlapping periods of 20 days confirm results obtained for visually determined waves. They show systematic inversions in spread rates between countries/regions, which occur approximately every 80–90 days, across the whole period studied. The causes for this are not well understood but might indicate compensation mechanisms for reduced spread rates during confinement in periods following the confinement. This reduction is shown and analyzed in many countries imposing a confinement like England, France, Germany, Iran, Italy, Netherlands, Spain, United States and China compared to countries like Sweden and South Korea, which did not implement mandatory stay-at-home confinements [39,40,41]. A stochastic modeling of the reducing effect of confinement is possible and shows a control depending on the characteristics of the countries concerned and on the early or late nature of this mitigation policy [42].

However, confinements might select for more contagious viral strains, and/or viral strains that are contagious for longer periods. The increased contagiousness and possibly pathogenicity [43,44,45] of these viruses would cause higher spread rates after deconfinement and therefore reduce the positive impact of restrictive measures such as the lockdown. This working hypothesis on effects of lockdowns arises from the results presented above and could be tested by ulterior analyses specifically designed to examine this working hypothesis.

## 5. Conclusions

In the USA, first and second wave slopes are not correlated with temperature, median age or time since wave onset, but with population density. The principle of inverted trends between first and second waves upholds; density effects are positive/negative for first/second wave slopes. Negative associations between population density and viral spread rates are also observed when examining countries across the world. Such negative associations of viral spread with population density are not compatible with our present understanding of the epidemiology of infectious agents. These inversions of directions of associations between viral spread rates and environmental and populational variables are confirmed by analyses of consecutive nonoverlapping periods of 20 days. We also observe that viral spread rates at any time during the study period are inversely proportional to rates 80-90 days later. These results were observed in two independent samples, US states and 51 countries across the globe. They stress that pandemic dynamics are misunderstood and probably mismanaged.

## Figures and Tables

**Table 1:** Web sites for data sources, accessed on 20 August 2020.

**Temperature**	https://www.currentresults.com/Weather/US/average-annual-state-temperatures.php
**Elevation**	https://en.wikipedia.org/wiki/List_of_U.S._states_and_territories_by_elevation
**Density**	https://en.wikipedia.org/wiki/List_of_states_and_territories_of_the_United_States_by_population_density
**Median age**	https://en.wikipedia.org/wiki/List_of_U.S._states_and_territories_by_median_age.

**Table 2:** Spread parameters of first and second waves in the 50 states of the USA and the district of Columbia.

State	T	E	D	A	Date 1	Slope 1	Date 2	Dis	Slope 2
Alabama	17.1	150	37	39.2	21.03	0.3338			
Alaska	−3	580	1	34.6	16.03	0.175	23.05	7	0.051
Arizona	15.7	1250	23	37.9	22.03	0.1841	25.05	6	0.0733
Arkansas	15.8	200	22	38.3	19.03	0.0967	10.05	8	0.0502
California	15.2	880	95	36.8	14.03	0.1662	10.05	−4	0.0223
Colorado	7.3	2070	20	36.9	20.03	0.1989	11.06	2	0.0408
Connecticut	9.4	150	286	41	18.03	0.2759			
DC		50		34	21.03	0.1011	30.06	6	0.0719
Delaware	12.9	20	187	40.7	22.03	0.1463	6.06	−6	0.0412
Florida	21.5	105	145	42.2	15.03	0.2584	26.05	−11	0.0785
Georgia	17.5	180	68	36.9	15.03	0.2536	25.05	0	0.0293
Hawaii	21.1	920	86	39.2	23.03	0.1433	2.05	−21	0.0906
Idaho	6.9	1520	7	36.6	24.03	0.2793	5.06	−3	0.0991
Illinois	11	180	89	38.3	16.03	0.4308	15.06	1	0.0289
Indiana	10.9	286	71	37.9	24.03	0.1093	22.06	1	0.0348
Iowa	8.8	340	21	38.2	24.03	0.1017	16.04	2	0.0893
Kansas	12.4	610	14	36.9	23.03	0.0876	12.04	1	0.096
Kentucky	13.1	230	43	38.9	23.03	0.1743	10.6	−7	0.0344
Louisiana	19.1	30	41	37.7	15.03	0.2022	23.05	−6	0.0382
Maine	5	180	16	44.9	21.03	0.1092	18.04	0	0.0373
Maryland	12.3	110	238	38.8	25.03	0.1216	14.04	0	0.0127
Massachusetts	8.8	150	336	39.4	13.03	0.3015	15.06	−33	0.0129
Michigan	6.9	270	67	39.8	18.03	0.4173	15.06	2	0.0612
Minnesota	5.1	370	26	38.1	20.03	0.1194	14.04	−6	0.1172
Mississippi	17.4	90	24	37.7	20.03	0.0863			
Missouri	12.5	240	34	38.7	21.03	0.1762	29.05	14	0.0307
Montana	5.9	1040	2	39.9	14.03	0.2485	5.06	4	0.0965
North Carolina	15	210	79	38.9	19.03	0.1458	4.05	0	0.0272
North Dakota	4.7	580	4	35.2	27.03	0.0545	8.05	7	0.0914
Nebraska	9.3	790	9	36.6	29.03	0.0775	19.06	−7	0.0248
Nevada	9.9	1680	10	38.1	18.03	0.1992	23.06	−2	0.054
New Hampshire	6.6	300	57	43	21.03	0.1426			
New Jersey	11.5	80	470	40	16.03	0.3797			
New Mexico	11.9	1740	17	38.1	24.03	0.1126	9.06	−5	0.0347
New York	7.4	300	162	39	10.03	0.5503			
Ohio	10.4	260	109	39.4	19.03	0.2369	15.04	1	0.2485
Oklahoma	15.3	400	22	36.7	24.03	0.1267	8.06	2	0.1285
Oregon	9.1	1010	16	39.4	19.03	0.106	17.05	−9	0.0564
Pennsylvania	9.3	340	110	40.8	17.03	0.2935	14.06	1	0.0472
Rhode Island	10.1	60	394	40.1	21.03	0.146	24.05	1	0.0435
South Carolina	16.9	110	62	39.6	18.03	0.1536	24.05	0	0.0609
South Dakota	7.3	670	4	37.1	29.03	0.1716			
Tennessee	14.2	270	61	38.8	17.03	0.2258			
Texas	18.2	520	40	34.8	17.03	0.241	20.4	1	0.0281
Utah	9.2	1860	14	31	19.03	0.1146	11.05	−6	0.0329
Vermont	6.1	300	26	42.8	22.03	0.0546			
Virginia	12.8	290	81	38.4	19.03	0.1789	15.06	0	0.0173
West Virginia	11	460	29	42.7	23.03	0.0985	5.06	1	0.0565
Washington	9.1	520	41	37.2	14.03	0.0657	14.04	0	0.0494
Wisconsin	6.2	320	41	39.6	18.03	0.0910	12.04	0	0.0200
Wyoming	5.6	2040	2	38	28.03	0.2346	29.05	0	0.0646
P K-S	0.907	0.008	0.007	0.530		0.274			0.228

Columns are: 1. State, 2. Mean annual temperature, 3. Mean elevation, 4. Population density, 5. Median age, 6. Onset date of first wave, 7. Exponential slope of first wave, 8. Onset date of second wave, 9. Days separating onset date in column 8 vs. onset date determined by statistical analysis. P K-S is *p*-values along the Kolmogorov–Smirnov test for normality.

**Table 3:** Spread rates (×100) for COVID-19 in the 50 states of the USA and the district of Columbia for 14 consecutive nonoverlapping 20-day time periods. N > 0 are numbers of positive rates, Mean indicates mean rates. Days are since pandemic onset in that state, which differs among states. P K-S are *p*-values along the Kolmogorov–Smirnov test for normality.

State\Day	20	40	60	80	100	120	140	160	180	200	220	240	260	280
Alabama	24.82	0.51	2.67	4.61	6.49	5.17	−0.59	−4.30	−0.26	2.37	0.88	1.71	1.07	2.13
Alaska	14.70	−8.70	−2.76	14.64	0.52	6.41	5.18	0.60	0.35	1.64	2.81	3.03	0.29	−4.71
Arizona	28.61	−0.07	2.09	1.79	8.31	1.97	−0.58	−6.02	−3.77	3.92	4.05	4.03	6.06	2.25
Arkansas	23.24	1.66	−4.17	12.99	3.53	−0.57	0.39	−1.08	1.24	1.19	−0.71	−0.15	0.72	1.30
California	26.21	0.06	0.55	2.83	1.54	3.88	0.30	0.94	−1.79	−0.50	−0.06	3.14	7.85	6.02
Colorado	23.83	−0.17	0.42	−2.01	−4.76	2.25	1.56	−2.61	0.59	5.25	2.59	5.33	1.36	−2.47
Connecticut	32.48	3.46	−3.92	−3.20	−4.99	3.62	3.10	1.25	5.22	−1.33	2.86	4.44	0.24	−0.64
DC	18.43	1.18	2.00	−2.08	−4.00	2.50	1.52	−1.83	−1.70	0.98	4.37	4.06	2.96	1.71
Delaware	28.25	−4.30	9.34	−4.42	−2.68	5.02	−0.38	0.44	−0.16	1.33	2.04	2.78	5.10	3.54
Florida	22.08	−2.26	−0.88	−0.08	7.87	5.21	−1.21	−3.72	−1.81	0.11	1.26	3.72	2.18	1.86
Georgia	25.76	−0.58	0.03	0.60	2.28	5.68	0.41	−1.63	−1.06	−1.14	−0.25	1.87	2.48	4.07
Hawaii	22.85	−7.89	−9.60	−3.84	16.15	5.10	3.19	6.53	0.84	−3.67	−2.12	0.70	−0.43	1.59
Idaho	32.63	−9.28	−5.88	10.37	4.65	6.68	−0.40	−1.76	−1.81	4.39	1.93	1.50	1.40	−0.50
Illinois	23.16	1.72	2.51	−1.82	−5.17	1.47	1.68	0.61	0.53	0.47	2.26	4.61	−0.67	−0.52
Indiana	34.25	0.06	0.50	−0.26	−0.38	3.05	1.49	−0.26	0.08	0.83	3.39	4.90	1.09	0.23
Iowa	26.93	6.86	1.39	−2.82	−0.96	4.40	1.35	0.95	−1.62	2.78	2.55	6.94	−4.71	−1.99
Kansas	24.21	1.49	3.91	−4.31	3.20	5.06	−3.57	−2.44	−2.71	1.11	2.23	0.11	2.03	−1.89
Kentucky	23.89	4.07	0.83	−1.77	−3.16	3.47	2.67	−0.02	1.91	1.60	0.86	2.50	2.57	0.39
Louisiana	29.89	−7.88	−2.11	−0.57	2.96	9.53	0.65	−2.44	0.31	−1.71	−4.58	−0.96	4.92	1.24
Maine	22.35	−0.72	5.22	2.02	−3.99	−2.01	1.06	−1.42	0.17	2.29	0.41	7.93	1.97	5.99
Maryland	30.02	5.99	2.67	−1.11	−5.58	0.26	3.50	−1.58	0.94	−1.87	1.25	2.11	3.17	1.53
Massachusetts	33.10	2.80	−2.26	−3.74	−8.44	0.53	2.26	−1.50	0.58	1.50	1.08	3.73	1.75	2.75
Michigan	37.94	−4.00	−2.67	−2.12	−4.92	3.78	1.31	−0.70	0.28	0.30	2.46	5.67	−0.76	−5.05
Minnesota	19.01	4.60	10.34	0.17	−2.17	2.23	0.68	−0.94	2.25	2.73	1.34	5.57	1.74	−4.48
Mississippi	32.25	4.00	0.99	2.18	0.03	4.08	3.05	−2.57	0.33	−1.73	0.05	−2.05	0.65	1.93
Missouri	27.29	−0.48	−2.61	0.99	0.45	2.81	3.59	4.79	−0.07	1.11	1.81	2.43	−2.17	−2.36
Montana	23.86	−6.12	−12.88	0.37	1.80	6.77	2.18	1.47	3.11	2.09	5.24	1.53	2.02	0.28
Nebraska	19.14	6.55	4.39	0.15	−3.17	1.24	2.96	−1.57	−0.87	3.00	3.52	5.69	−3.28	−4.55
Nevada	26.55	−0.48	−1.13	1.49	3.87	5.54	1.94	−2.36	−3.26	−0.86	0.93	1.23	3.48	1.25
New Hampshire	21.28	0.15	1.25	−1.06	−5.27	−0.77	−0.26	−2.45	2.87	−1.76	3.97	4.10	3.68	2.58
New Jersey	37.46	0.15	−3.57	−2.64	−4.82	−0.34	0.72	1.42	1.39	2.89	3.09	4.41	2.64	0.98
New Mexico	14.28	2.46	1.26	−1.04	−1.64	3.72	1.49	−3.76	−1.33	4.51	5.04	2.87	3.75	−1.11
New York	24.05	−2.39	−3.75	−2.75	−3.60	−1.07	−1.39	0.34	2.43	−0.17	2.13	2.71	2.96	1.72
North Carolina	29.43	1.70	2.24	2.55	2.25	1.67	−0.78	−4.92	−0.54	1.87	1.86	1.48	3.16	3.14
North Dakota	16.30	6.56	−1.08	−3.77	−1.37	6.40	1.57	0.54	1.57	4.27	3.21	4.11	−1.41	−4.33
Ohio	28.97	5.10	−1.13	−0.19	0.40	2.70	−0.40	−1.75	2.29	−1.13	2.33	3.81	3.65	1.40
Oklahoma	28.27	−3.56	0.02	−1.77	7.42	3.31	2.65	−2.22	0.92	2.81	0.65	1.26	0.55	0.97
Oregon	20.27	−1.79	1.86	−4.64	7.45	2.49	0.80	−0.93	−0.16	1.03	0.64	3.44	2.56	−0.62
Pennsylvania	36.04	0.85	0.71	−2.20	−2.91	3.20	1.79	−0.87	1.90	0.38	2.64	3.76	3.93	2.27
Rhode Island	22.39	3.75	−2.60	−4.36	−4.45	−0.31	4.36	0.15	−2.40	2.90	4.50	3.31	1.90	−3.86
South Carolina	25.64	−1.69	0.97	1.62	5.17	2.57	−1.47	−3.29	2.79	−0.94	−0.61	0.54	2.35	4.14
South Dakota	28.01	5.63	3.39	−1.53	−1.63	0.91	3.10	1.72	0.78	5.42	5.22	2.97	−1.94	−2.23
Tennessee	26.84	6.02	0.08	0.87	1.11	5.39	2.35	−1.62	0.78	0.35	2.85	0.38	−0.82	3.97
Texas	27.73	0.05	2.81	−0.58	4.55	4.74	−1.22	−2.03	−2.72	1.57	2.34	4.11	1.29	3.50
Utah	27.54	0.26	0.87	0.11	1.53	0.46	−1.55	−1.20	1.68	4.67	0.97	1.45	0.94	−0.63
Vermont	22.07	−8.53	−0.13	8.59	−2.92	−2.88	−3.21	8.34	1.07	−1.86	9.30	4.99	9.21	3.59
Virginia	23.59	3.99	2.08	1.06	−4.81	0.55	1.80	−0.06	1.31	−1.40	1.70	1.57	3.72	3.33
W Virginia	26.40	−2.56	−1.81	1.89	4.96	8.56	1.41	0.19	3.71	1.37	3.18	4.71	1.54	−0.02
Washington	8.22	−6.72	1.03	0.59	0.89	3.15	0.85	−1.31	−2.75	2.27	2.45	4.66	2.43	0.46
Wisconsin	23.99	0.01	4.04	4.10	−0.82	3.90	0.40	−0.82	1.67	4.20	0.65	2.05	−3.06	−1.76
Wyoming	17.98	−3.48	8.95	0.17	9.48	3.00	3.04	−4.67	−2.25	5.93	3.27	3.86	1.90	−3.35
N > 0	51	29	32	24	26	44	37	16	31	37	45	48	41	31
Mean	25.38	−0.038	0.323	0.316	0.397	3.069	1.086	−0.831	0.253	1.321	2.075	3.033	1.765	0.491
P K-S	0.776	0.399	0.529	0.113	0.754	0.989	0.883	0.545	0.867	0.920	0.794	0.790	0.654	0.670

**Table 4:** Spread rates (×100) for COVID-19 in the 51 countries for 15 consecutive nonoverlapping 20-day time periods. N > 0, numbers of positive rates, Mean is mean rates. Days are since pandemic onset in that state, which differs among states. P K-S are *p* values along the Kolmogorov–Smirnov test for normality.

Country\Day	20	40	60	80	100	120	140	160	180	200	220	240	260	280	300
Afghanistan	15.28	5.00	7.26	5.32	−1.29	−2.26	−0.48	−6.00	−6.08	−1.22	11.19	1.60	4.80	0.36	
Albania	14.49	−0.84	−5.79	−2.20	5.02	0.97	1.30	1.88	−1.00	1.65	0.89	2.66	4.13	1.11	
Armenia	16.65	−0.79	5.72	7.07	2.12	−0.68	−4.33	−0.87	0.48	1.45	3.92	1.69	−4.83	−3.31	−5.01
Australia	25.07	−4.71	−11.82	−0.04	0.83	11.06	9.07	2.56	−7.88	−4.05	−7.59	−0.08	−3.59	−1.24	7.08
Austria	30.23	4.16	−10.29	−4.76	−6.94	1.50	5.69	2.13	5.72	0.12	2.43	4.22	7.34	−2.42	−2.19
Bangladesh	5.26	24.62	4.00	3.71	2.10	−0.57	−0.49	1.25	−2.11	−1.02	0.53	1.10	2.44	−1.72	
Belgium	29.94	5.88	−2.79	−3.27	−5.26	−1.73	1.74	6.97	−0.49	2.05	3.52	9.38	−8.02	−4.66	−0.16
Bolivia	14.65	7.18	8.70	5.72	1.63	2.12	1.10	0.23	−2.76	−0.96	−2.08	−1.72	1.90	6.46	
Bosnia	18.43	4.10	−0.38	−5.19	5.36	6.15	0.90	0.32	1.22	−0.42	−2.27	7.65	−2.09	−2.28	−3.57
Brazil	33.73	8.57	5.18	6.82	2.17	2.05	−0.93	1.46	−0.73	−2.52	−0.86	0.53	1.65	2.28	0.04
Canada	25.04	14.58	1.87	−1.82	−2.58	−4.04	0.10	1.97	0.88	2.09	4.29	1.54	2.68	1.28	1.28
Chile	31.06	6.44	1.73	5.41	1.47	−1.95	−2.31	−0.97	−1.28	0.70	1.06	0.00	2.05	0.88	1.54
Czechia	30.07	2.34	−6.20	−1.42	−2.70	2.91	−4.90	1.87	0.34	8.70	2.59	8.27	−2.65	−1.15	5.81
Georgia	7.56	5.78	−0.16	0.08	2.65	−6.34	3.13	−6.75	−2.31	15.53	6.30	8.63	4.03	0.63	−6.27
Germany	35.16	7.43	−5.98	−5.09	−5.06	3.36	−2.52	4.94	4.69	−2.04	2.07	6.73	4.30	0.30	3.30
Greece	18.28	−1.52	−3.81	−2.05	6.38	0.93	1.25	12.28	1.55	0.62	0.30	5.47	2.79	−3.74	−4.50
Hungary	19.48	6.03	−0.99	−3.38	−7.37	−1.73	6.18	1.61	2.73	10.63	1.61	4.88	3.25	1.92	−4.47
India	19.78	14.27	4.25	5.48	3.25	3.71	3.24	2.23	0.40	1.75	−0.67	−1.73	0.01	−0.20	−2.43
Indonesia	31.30	6.62	0.21	3.57	3.00	1.29	0.54	0.85	2.18	1.26	−0.09	−0.51	2.31	1.57	1.37
Iran	40.40	6.66	−4.41	−0.19	1.85	−0.87	0.10	0.02	−0.48	−1.72	2.88	1.14	4.16	2.47	−2.79
Iraq	14.82	8.94	−5.01	3.21	9.96	3.94	1.20	2.04	3.05	0.49	−0.95	−1.32	−0.96	−2.68	−2.93
Ireland	30.39	6.56	−5.86	−7.61	−10.15	−4.52	4.66	9.26	1.82	4.31	3.79	3.43	−3.89	−1.98	8.65
Israel	28.61	12.23	−2.47	−11.53	12.25	5.22	7.81	−0.06	1.18	3.82	1.80	−10.38	0.55	1.70	3.79
Italy	35.43	6.45	−1.99	−5.16	−5.06	−2.65	−2.39	1.62	4.41	6.08	0.35	7.27	6.76	−0.68	−2.62
Kazakhstan	21.10	5.98	0.55	6.71	−0.22	8.67	−0.94	−6.96	−7.08	−1.47	2.07	9.07	1.27	−0.50	
Kuwait	4.17	6.19	3.04	7.45	−0.56	−2.01	0.33	0.15	1.49	0.99	−1.78	2.36	−0.29	−4.74	−1.18
Lithuania	21.70	−3.11	−9.79	−2.26	−0.20	2.46	6.22	4.34	−0.17	5.18	1.44	8.25	0.67	1.40	
Luxemburg	29.22	−8.42	−6.55	−6.81	0.46	14.02	5.36	−5.47	0.30	2.60	4.25	7.57	−2.28	0.16	
Malaysia	31.13	1.47	−6.38	−2.08	4.30	−3.72	2.53	3.78	3.42	6.77	4.92	5.48	0.82	1.23	2.00
Mexico	26.38	8.11	4.82	3.36	1.52	0.79	0.89	−0.24	0.39	−1.99	1.88	0.09	−0.83	1.38	0.45
Moldova	19.26	1.80	0.03	1.23	4.22	−4.23	0.68	1.60	0.83	−0.64	0.09	−0.30	1.41	−0.78	
Morocco	25.02	6.01	−1.31	−5.95	4.98	6.50	4.17	3.80	−0.23	2.65	2.04	0.16	−1.43	−1.66	
N Macedonia	19.60	5.10	−8.86	1.80	10.53	0.38	0.31	−0.69	−4.68	3.92	5.90	5.55	1.80	−3.23	
Netherlands	33.96	6.11	−1.67	−4.50	−2.12	−6.07	−1.05	8.25	1.24	2.96	6.15	4.91	−3.62	−0.79	5.22
Norway	32.24	3.04	−4.34	−4.61	−5.32	−4.10	−8.34	10.91	2.80	5.89	−0.32	1.04	5.25	−2.75	1.26
Pakistan	32.05	5.03	6.72	2.79	7.68	−2.83	−5.12	−5.37	−3.39	2.12	−0.64	3.03	4.91	0.68	−3.49
Panama	25.65	2.63	0.32	1.01	4.09	1.48	−1.03	−0.60	0.20	−0.35	1.03	1.06	4.05	3.42	1.44
Philippines	23.02	1.90	−0.50	−0.89	3.50	3.59	2.59	5.20	−1.10	0.90	−1.60	−2.30	−1.77	−0.62	0.97
Poland	32.90	6.28	−0.37	1.45	1.00	−2.38	0.93	4.22	1.28	−3.03	6.77	7.00	1.16	−4.49	−0.04
Portugal	32.57	3.86	−4.33	−1.82	2.48	1.43	0.19	−2.99	−0.43	4.72	1.36	7.84	3.59	−4.45	−1.16
Romania	25.79	3.61	−0.81	−4.61	0.44	1.88	5.16	0.03	−0.50	1.01	3.58	2.86	1.94	−1.54	−3.23
Russia	24.49	15.79	5.52	−0.90	−0.10	−1.62	−0.46	−0.76	−0.46	0.81	3.51	1.89	1.35	1.18	0.40
S Korea	39.90	−5.49	−8.29	−5.98	7.20	3.22	0.91	−2.40	−3.56	13.72	−5.90	−0.99	1.88	5.77	3.62
Saudi Arabia	26.61	6.47	6.99	3.68	1.52	−0.50	−2.70	−3.71	−1.80	−2.88	−2.03	−0.83	−1.19	−1.99	0.01
Serbia	24.03	8.49	−5.76	−1.45	5.40	9.67	1.79	−3.79	−6.40	0.43	4.49	14.24	7.72	0.85	−3.55
Turkey	42.42	3.06	−4.63	−2.49	3.35	−1.02	−0.54	1.67	1.82	0.18	0.31	1.41	6.30	1.44	
UAE	5.12	16.42	1.89	2.99	−2.70	−0.51	−2.32	−1.13	3.67	4.10	2.32	1.31	−0.34	0.03	0.45
UK	35.66	14.62	0.24	−1.45	−4.09	−2.13	−3.28	0.73	2.28	4.96	4.96	3.90	0.68	−3.83	4.09
Ukraine	27.04	10.09	−0.42	−0.49	3.69	−0.96	2.53	2.75	1.24	1.99	2.88	3.10	2.34	−1.29	
USA	35.93	24.23	0.89	−0.29	−0.67	0.17	3.35	0.60	−0.80	−1.64	0.54	0.79	3.63	2.38	1.99
Uzbekistan	14.33	1.90	1.02	3.81	4.71	4.62	1.32	−0.89	−3.71	2.43	−2.68	−2.47	−1.17	1.02	
N > 0	51.00	44.00	21.00	21.00	33.00	27.00	33.00	33.00	27.00	36.00	37.00	39.00	35.00	25.00	21.00
Mean	25.15	6.02	−1.20	−0.35	1.35	0.88	0.85	1.06	−0.15	2.03	1.58	2.87	1.31	−0.33	0.14
P K-S	0.730	0.109	0.927	0.871	0.880	0.657	0.532	0.324	0.430	0.224	0.994	0.599	0.933	0.962	0.868

**Table 5:** Pearson correlation coefficients r (×100) between spread rates. Above the diagonal, consecutive nonoverlapping 20-day periods from the USA (Table 3), and, below the diagonal, from various countries (Table 4). Highlights are for *p* < 0.05, two-tailed test.

Period	20	40	60	80	100	120	140	160	180	200	220	240	260	280
20		8.44	−20.28	−14.06	−12.91	2.06	−5.35	1.08	19.97	−20.66	−16.90	−13.99	1.25	22.38
40	−12.19		34.97	−35.06	−41.88	−27.16	19.09	−20.75	8.95	11.97	9.16	12.29	−32.21	−10.46
60	−30.70	46.88		−13.30	−14.46	−21.45	−17.35	−37.87	−25.24	27.82	4.15	26.25	5.78	−1.48
80	−43.48	19.60	60.91		18.56	3.12	−7.80	0.98	−3.24	2.78	1.03	−19.44	9.14	7.52
100	−16.05	−12.48	2.74	14.58		45.82	−16.25	−15.34	−29.57	−0.86	−34.56	−38.75	0.67	10.38
120	−3.47	−28.73	−29.64	−9.71	30.60		10.69	−14.23	−11.02	−2.38	−31.61	−31.53	−16.50	−14.27
140	−10.69	−13.90	−38.06	−35.52	10.33	39.24		16.86	10.11	6.74	−2.21	3.73	−42.42	−38.85
160	18.81	−3.84	−31.98	−36.01	−28.21	−25.49	4.07		33.33	−29.27	22.71	13.97	−7.00	−7.52
180	19.53	13.42	−11.88	−35.10	−34.54	−36.00	−9.64	49.67		−24.47	−2.14	−2.01	−10.99	0.32
200	−1.15	−18.88	−28.08	−37.32	−7.39	−25.39	1.72	−4.11	15.19		30.54	28.37	−32.57	−49.68
220	0.65	17.93	6.56	−8.7	−23.01	−35.36	−7.58	−8.67	10.00	11.19		46.64	10.23	−23.15
240	5.33	−19.27	−32.94	−13.92	−32.56	2.86	−9.33	−4.04	−3.33	19.67	40.71		−8.76	−22.56
260	15.67	14.91	−4.48	−3.11	4.91	−15.03	−14.76	−22.09	4.90	3.98	9.66	10.70		56.77
280	14.85	0.86	13.4	1.48	16.47	11.04	12.40	−29.26	−19.98	11.38	−20.61	−31.81	31.23	
300	43.39	2.23	−32.48	−29.66	−26.21	4.74	4.05	33.80	−4.15	−4.35	−10.73	−26.07	−35.84	12.88

**Table 6:** Pearson correlation coefficients r (×100) between spread rates for consecutive nonoverlapping 20-day periods from Table 3 and Table 4 with environmental variables from Table 2 and Table 6. Rows starting with USA give r between spread rates and environmental factors for the US states, rows starting with world are for countries. Rows starting with rs present nonparametric Spearman rank correlation coefficients rs for the two variables, altitude and density, that differ at *p* < 0.05 from normality according to Kolmogorov–Sminov tests. Bold, underlined indicate that correlations are statistically significant at *p* < 0.05 according to a two- and one-tailed test, respectively.

Variable\Day	20	40	60	80	100	120	140	160	180	200	220	240	260	280
Temp. USA	25.12	4.70	−7.16	−16.21	**47.06**	21.18	−21.98	−19.91	−21.60	−40.10	**−50.69**	**−49.46**	16.29	**51.98**
World	−19.04	4.51	23.03	**34.31**	**31.66**	15.94	7.09	−6.62	−5.28	−23.11	**−38.76**	**−38.72**	−10.68	13.34
Altitude USA	−23.09	−18.78	−1.66	6.53	**32.71**	11.81	1.28	−19.04	**−29.20**	**49.98**	14.48	1.34	3.90	**−28.26**
rs	−4.8	−19.9	1.5	12.8	**32.31**	3.85	−8.56	0.52	−6.69	**42.04**	21.23	6.97	−1.97	−23.1
World	−11.64	−10.61	**29.92**	**33.46**	18.73	−3.51	−13.12	**−35.17**	**−30.11**	−5.92	7.80	−24.34	12.08	22.81
rs	−4.9	−8.9	22.88	19.79	27.61	6.34	−5.22	**−36.68**	**−32.67**	−13.16	−1.60	−21.75	26.36	25.92
Density USA	**38.85**	17.62	−17.62	**−36.69**	**−41.00**	**−31.03**	14.32	13.56	14.52	−18.43	3.41	10.16	14.58	12.67
rs	11.54	26.24	−10.30	**−36.64**	**−34.65**	−5.49	7.61	16.86	12.70	**−38.32**	−9.86	10.74	8.77	20.96
World	−19.90	24.74	6.45	−4.70	4.56	−4.78	1.62	10.29	7.00	13.47	−13.05	−10.58	−11.70	−14.60
rs	8.63	−5.25	−20.88	−25.96	7.27	0.91	4.33	19.35	**32.83**	**36.69**	0.22	12.44	−10.41	−23.4
Age USA	15.17	−9.38	−5.15	−7.11	−13.10	−21.44	−7.13	17.25	**32.06**	**−33.31**	11.68	25.91	21.84	**30.23**
World	**33.71**	−16.10	**−50.50**	**−54.53**	**−39.52**	−2.62	9.08	**30.14**	25.74	**29.10**	12.27	57.83	7.62	−22.37

**Table 7:** Environmental variables for 51 countries, mean annual temperature, mean elevation, population density per square kilometer, and median age. P K-S is the *p*-value along the Kolmogorov–Smirnov test for normality.

Country	Temperature	Elevation	Density	Age
Afghanistan	12.6	1885	49	18.9
Albania	11.4	708	99.73	32.9
Armenia	7.15	1792	99	35.1
Australia	21.65	330	3	38.7
Austria	6.35	910	106	44
Bangladesh	25	85	1175	26.7
Belgium	9.55	181	378	41.4
Bolivia	21.55	1192	10	24.3
Bosnia	9.85	500	69	42.1
Brazil	24.95	320	25	32.6
Canada	−5.35	487	4	42.2
Chile	8.45	1871	23	34.4
Czech	7.55	433	135	42.1
Georgia	5.8	1432	53.51	38.1
Germany	8.5	263	233	47.1
Greece	15.4	498	210	44.5
Hungary	9.75	143	272	42.3
India	23.65	621	412	28.1
Indonesia	25.85	367	141	30.2
Iran	17.25	1305	51	30.3
Iraq	14.03	312	90	20
Ireland	9.3	118	70	36.8
Israel	19.2	508	417	29.9
Italy	12.45	538	200	45.5
Kazakhstan	6.4	387	7	30.6
Kuwait	25.35	108	642	29.3
Lithuania	6.2	110	73	43.7
Luxemburg	8.65	325	237.39	39.3
Malaysia	25.4	538	99	28.5
Mexico	21	1111	64	28.3
Moldova	9.45	139	79.27	36.7
Morocco	17.1	909	80	29.3
N Macedonia	9.8	741	81	37.9
Netherlands	9.25	30	421	42.6
Norway	1.5	460	17	39.2
Pakistan	20.2	900	274	23.8
Panama	25.4	360	56	29.2
Philippines	25.85	442	362	23.5
Poland	7.85	173	123	40.7
Portugal	15.15	372	112	42.2
Romania	8.8	414	81.4	41.1
Russia	−5.1	600	9	39.6
S Korea	11.5	282	517	41.8
Saudi Arabia	24.65	665	16	27.5
Serbia	10.55	473	89.08	42.6
Turkey	11.1	1132	106.12	30.9
UAE	27	149	116.87	30.3
UK	8.45	162	280	40.5
Ukraine	8.3	175	69.49	40.6
USA	8.55	760	34	38.1
Uzbekistan	12.05	2750	73	28.6
P K-S	0.199	0.024	0.002	0.259

## Data Availability

All the data are obtained from readily available public databases that are cited in the text.
